# Evolving treatment paradigms for PCV

**DOI:** 10.1038/s41433-021-01688-7

**Published:** 2021-07-14

**Authors:** Beau J. Fenner, Chui Ming Gemmy Cheung, Shaun S. Sim, Won Ki Lee, Giovanni Staurenghi, Timothy Y. Y. Lai, Paisan Ruamviboonsuk, Gregg Kokame, Yasuo Yanagi, Kelvin Y. C. Teo

**Affiliations:** 1 Singapore National Eye Centre and Singapore Eye Research Institute, Singapore, Singapore; 2 Duke-NUS Graduate Medical School, National University of Singapore, Singapore, Singapore; 3 Nune Eye Hospital, Seoul, South Korea; 4 Eye Clinic, Department of Biomedical and Clinical Sciences “Luigi Sacco”, University of Milan, Milan, Italy; 5 Department of Ophthalmology and Visual Sciences, The Chinese University of Hong Kong, Hong Kong Eye Hospital, Hong Kong, China; 6 Department of Ophthalmology, Rajavithi Hospital, Bangkok, Thailand; 7 Division of Ophthalmology, Department of Surgery, University of Hawaii School of Medicine, Honolulu, HI USA; 8 Department of Ophthalmology and Microtechnology, Yokohama City University, Yokohama, Japan; 9 University of Sydney, Sydney, Australia

**Keywords:** Macular degeneration, Retinal diseases

## Abstract

Polypoidal choroidal vasculopathy (PCV) is a subtype of neovascular AMD (nAMD) that accounts for a significant proportion of nAMD cases worldwide, and particularly in Asia. Contemporary PCV treatment strategies have closely followed those used in typical nAMD, though there are significant gaps in knowledge on PCV management and it remains unclear if these strategies are appropriate. Current clinical trial data suggest intravitreal anti-vascular endothelial growth factor (VEGF) therapy alone or in combination with photodynamic therapy is effective in managing haemorrhage and exudation in PCV, although the optimal treatment interval, including as-needed and treat-and-extend approaches, is unclear. Newer imaging modalities, including OCT angiography and high-resolution spectral domain OCT have enabled characterisation of unique PCV biomarkers that may provide guidance on how and when treatment and re-treatment should be initiated. Treatment burden for PCV is a major focus of future therapeutic research and several newly developed anti-VEGF agents, including brolucizumab, faricimab, and new modes of drug delivery like the port delivery system, offer hope for dramatically reduced treatment burden for PCV patients. Beyond anti-VEGF therapy, recent developments in our understanding of PCV pathophysiology, in particular the role of choroidal anatomy and lipid mediators in PCV pathogenesis, offer new treatment avenues that may become clinically relevant in the future. This article explores the current management of PCV and more recent approaches to PCV treatment based on an improved understanding of this unique disease process.

## 摘要

息肉状脉络膜血管病变 (Polypoidal choroidal vasculopathy, PCV)为新生血管性AMD (neovascular AMD, nAMD) 的亚型, 患病人数在全球尤其是亚洲占相当大的比例。目前PCV的治疗策略密切遵循典型nAMD的治疗策略, 尽管在PCV管理方面存在巨大差距, 但仍不清楚这些策略是否适当。目前的临床试验数据表明, 单独行玻璃体内抗血管内皮生长因子 (vascular endothelial growth factor, VEGF) 或联合光动力疗法可有效管理PCV的出血和渗出, 但最佳治疗间隔 (包括按需治疗和延长治疗时间) 尚不清楚。包括OCT血管成像和高分辨率频域OCT在内的新型成像方式已能够表征PCV独特的生物标志物, 这可为如何以及何时开始治疗和再治疗提供指导。PCV的治疗负担是未来治疗研究的主要焦点, 新开发的brolucizumab、faricimab等抗VEGF药物, 以及如缓释给药系统等新的给药模式, 为显著降低PCV患者的治疗负担提供了希望。除抗VEGF治疗外, 我们对PCV病理生理学认识的最新进展, 尤其是脉络膜解剖和脂质介质在PCV发病机制中的作用, 提供了可能在未来具有临床相关性的新治疗途径。本文基于对这一独特疾病过程的深度理解, 探讨了目前PCV的管理和治疗的最新方法。

## Introduction

Age-related macular degeneration (AMD) is a leading cause of irreversible vision loss worldwide [1, 2]. Several subtypes of neovascular AMD (nAMD) can be characterised based on multimodal imaging [3]. Polypoidal choroidal vasculopathy (PCV), while currently considered a subtype of nAMD, is more common in Asian populations, comprising up to 50% of nAMD cases presenting to retina specialist clinics [4–7]. Morphologically, the lesion complex in PCV consists of polypoidal lesions (PL) with an associated branching neovascular network (BNN) best detected using indocyanine green angiography (ICGA) [8–10]. In addition to clinical features, differences in the pathogenesis of PCV and typical nAMD have also been proposed. For example, drusen and pigmentary changes which are considered the hallmark of early AMD are not commonly seen in PCV. In contrast, a congested choroidal background and association with a history of central serous chorioretinopathy have been reported to be common in PCV.

Despite these differences, current therapeutic options of PCV have largely evolved from treatments designed for typical nAMD. In this review we will discuss how the latest clinical trials in PCV, coupled with improved understanding of pathogenesis from imaging studies, have progressively contributed to the formulation of PCV-specific treatment approaches. We go on to highlight some emerging treatments that may soon play an important role in PCV management.

## Current PCV treatment options

Prior to the availability of optical coherence tomography, the PL was the main target of treatment, as this was the most obvious source of haemorrhage and exudation based on clinical examination, fluorescein angiography, and ICGA. Closure of the PL with focal laser or subsequently with verteporfin photodynamic therapy (PDT) have been widely used [11–15].

Historically, the management of PCV with PDT was complicated by acute vision loss and massive subretinal haemorrhage [16]. These complications were reduced with the introduction of anti-vascular endothelial growth factor (VEGF) therapies. The EVEREST trial [17] was one of the earliest PCV-specific randomised controlled trials. The sample size was limited and the primary endpoint was based on PL closure rate assessed with ICGA. This trial showed that PDT was superior to ranibizumab monotherapy in achieving PL closure (78% versus 29% after 6 months). With the results from the EVEREST study, PDT remained popular as the first choice of treatment in PCV. Subsequently, the LAPTOP trial [18] compared PDT monotherapy with ranibizumab monotherapy in PCV and reported superior visual outcome in the ranibizumab arm. However, PL closure rate was not reported and this has been criticised by some as a weakness of the study at the time of reporting [19]. However, the findings from the LAPTOP trial did serve to confirm a prevailing hypothesis that good functional outcomes are achievable even without PDT, and that PL closure may not necessarily be related to functional outcomes. In the EVEREST II study [20], ranibizumab combined with PDT achieved superior visual gains compared to ranibizumab alone (8.3 versus 5.1 ETDRS letters, at 12 months) while at the same time also achieving a higher rate of PL closure (69.3% versus 34.7%). These studies suggested a benefit of combining anti-VEGF therapy with PDT and has led to a decline in PDT monotherapy.

**Fig. 1:**
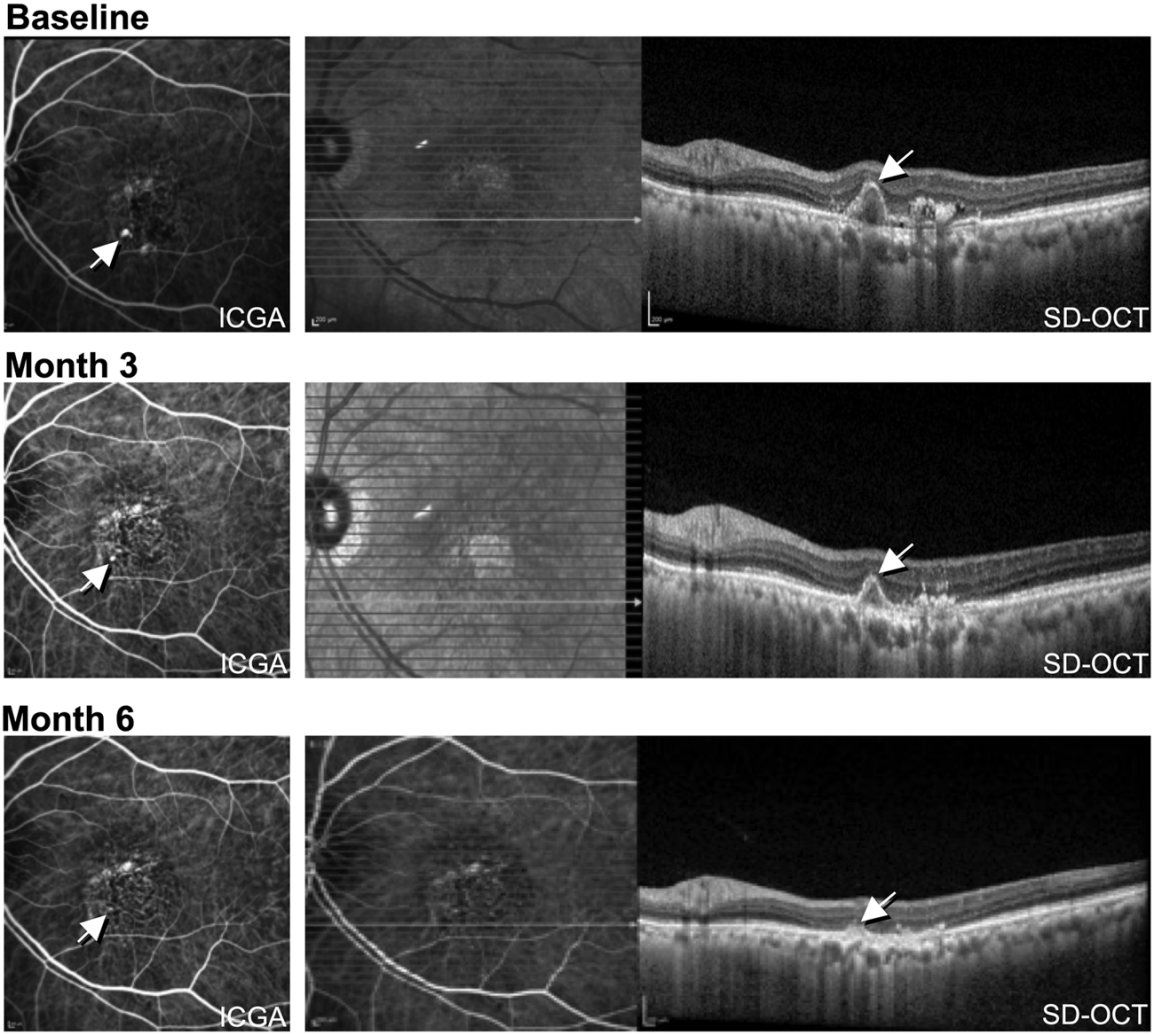
Demonstration of polyp closure in PCV following treatment with intravitreal aflibercept. Images were obtained using indocyanine green angiography (ICGA, shown on left) and spectral domain optical coherence tomography (SD-OCT, shown on right) at baseline, 3, and 6 months following treatment initiation. The polypoid lesion is indicated with an arrow, with progressive closure seen at 3 and 6 months.

The PLANET study [21, 22] evaluated the efficacy of intravitreal aflibercept in the management of PCV, either as monotherapy or with limited use of rescue PDT for patients unresponsive to aflibercept monotherapy. The primary endpoint demonstrated that the monotherapy arm was non-inferior to the combination arm (10.7 versus 10.8 ETDRS letter gains), although due to the strict criteria for the use of PDT, most of the combination therapy group (about 85% of patients) completed the study with only anti-VEGF monotherapy. In terms of anatomical outcome, most eyes (nearly 80%) had a fluid-free retina based on OCT at the end of the study, despite <40% of eyes achieving PL closure on ICGA (see Fig. 1). The PLANET study demonstrated the apparent efficacy and safety of anti-VEGF monotherapy in PCV and, in addition, the results suggested that good visual outcomes are compatible with the presence of an unclosed PL. The proposed ‘polyp inactivation’, characterised by absence of fluid despite PL persistence also initiated further research questioning the significance of PL closure.

Results of these therapeutic studies were also important in the evolving understanding of the nature of PCV lesions. While earlier studies emphasised the difference in the benefit of PDT in PCV compared to typical nAMD, the two lesions had been proposed to be distinct entities. With more recent studies which demonstrate that anti-VEGF therapy can be used successfully as first-line therapy in PCV and typical nAMD, the similarities of these two subtypes were increasingly appreciated. Concurrently most imaging and histopathology studies place the BNN in PCV lesions in a plane between the retinal pigment epithelium (RPE) and Bruch’s membrane. This finding further supports a conclusion that the BNN is a type 1 MNV instead of an intrachoroidal vascular channel as suggested in earlier studies based on ICGA, which did not have the benefit of depth resolution that OCT offers. There have also been some reports that PCV may uncommonly display a type 2 MNV appearance, affirming similar features to the typical nAMD subtype [23, 24].

## Anti-VEGF treatment regimens for PCV

Recognising that the BNN component of the PCV lesion is neovascular in nature, the anti-VEGF treatment regimens in PCV have also progressively become more aligned with that in typical nAMD. A popular treatment protocol for typical nAMD is the treat and extend (T&E) regimen. Briefly, the T&E protocol dictates that patients receive three initial monthly loading doses of anti-VEGF therapy followed by progressive adjustment of the treatment interval according to anatomical and functional outcomes. This interval can be extended by weeks or months with the aim of reducing treatment burden. These regimens use disease activity markers of fluid in retinal compartments, visual acuity and presence of haemorrhage on clinical examination for retreatment decisions. While not as extensively studied, PCV patients undergoing a T&E protocol show favourable outcomes comparable to patients with typical nAMD [25–31]. However, the current disease activity indicators used for guiding subsequent retreatment decisions are based on OCT features derived from experience in typical nAMD. It remains unclear if similar disease activity markers are sufficient for the optimal treatment of PCV. For example, recent evidence for more “relaxed” retreatment criteria for typical nAMD demonstrated comparable outcomes to a strict “fluid free” strategy. Here, the FLUID study demonstrated that up to 200 μm of subretinal fluid (SRF) at the fovea was well-tolerated over 2 years in nAMD cases, although it did not differentiate between nAMD subtypes [32]. However, given the prominence of SRF as a marker of disease activity in PCV, there is clearly a need to determine whether SRF is similarly well-tolerated in PCV. While some clinicians have already adopted the findings of the FLUID study into their practice for certain PCV patients, there is a need to confirm these findings with a controlled clinical trial with PCV patients. More recent work from several groups [33–35] demonstrates that fluctuations in retinal thickness results in poorer visual outcomes, and this finding, combined with the observation from EVEREST II that eyes managed with combination therapy had less persistent SRF [20], suggest that the FLUID study may be less applicable to PCV management.

**Fig. 2:**
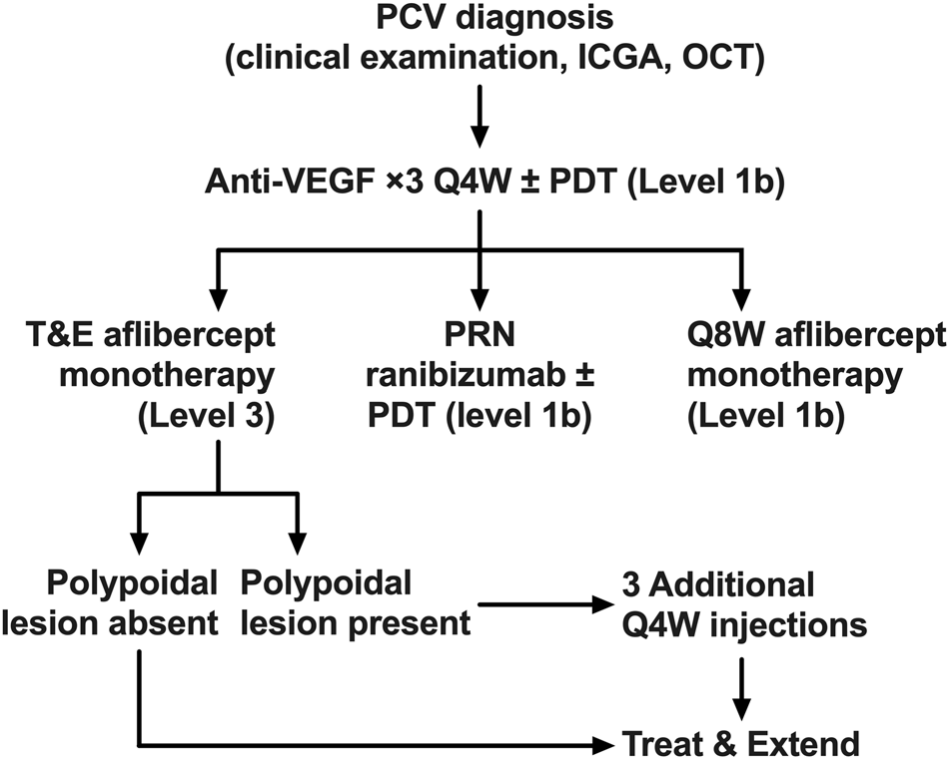
Treatment pathways for PCV. Anti-VEGF monotherapy can be used based on outcomes from the PLANET trial [21, 22], or combination therapy used based on outcomes from the EVEREST trials [17, 20, 99]. Alternatively, recent work suggests that PCV may be treated with potentially fewer injections and better functional outcomes using a treat-and-extend approach.

In addition to fluid, the PL within the sub-RPE vascular network is responsible for much of the exudation in PCV [9]. A potential limitation of current anti-VEGF treatment protocols is the lack of consideration of PL status. Chaikitmongkol et al. recently demonstrated a 50% complete polyp closure rate within 2 months following initiation of aflibercept [36]. Thus the speed of PL closure could be an additional consideration in titrating treatment. In a recent randomised controlled trial, eyes with persistent PL after three initial monthly loading injections of aflibercept that were treated with an additional three monthly injections achieved comparable visual gain and higher PL closure rate at month 6 and month 12 [37]. Addition of PDT is another alternative for eyes with residual PL. This was evaluated in the FUJISAN study [38], which showed that intravitreal ranibizumab combined with initial or deferred PDT had similar functional outcomes (8.1 versus 8.8 ETDRS letter gains in initial and deferred PDT arms, respectively, at 1 year). While the initial PDT arm required fewer additional ranibizumab injections compared to deferred PDT, only about half of the population in the deferred PDT arm required adjunct PDT. The outcomes of the above clinical trials are summarised in Table 1, and their practical application is illustrated in the treatment pathway shown in Fig. 2.

**Table 1:** Treatment outcomes in PCV treatment trials. Adapted from previous work [98].

Study	Follow-up (months)	Treatment	Patients in trial arm (n)	Number of injections	Number of PDTs	Polyp regression rate, %	Baseline visual acuity (ETDRS letters)	Mean vision change (ETDRS letters)
EVEREST (2012) [17]	6	Ranibizumab ×3 Q4W → PRN	21	5.2	1.9 (sham)	28.6	49.0	9.2
		Ranibizumab ×3 Q4W + PDT → PRN	19	3.9	1.7	77.8	57.2	10.9
EVEREST II (2017) [99]	12	Ranibizumab ×3 Q4W → PRN	168	7.3	2.3 (sham)	34.7	61.1	5.1
		Ranibizumab ×3 Q4W + PDT → PRN	154	5.2	1.5	69.3	61.2	8.3
EVEREST II (2020) [20]	24	Ranibizumab ×3 Q4W → PRN	168	12.0	**–**	26.7	61.1	5.5
		Ranibizumab ×3 Q4W + PDT → PRN	154	6.0	2.0	56.6	61.2	9.6
LAPTOP (2013) [18]	12	Ranibizumab ×3 Q4W → PRN	47	5.8	–	Not reported	88.0	4.0
		Initial PDT → PRN	46	5.2	1.5	Not reported	84.0	−2.0
FUJISAN (2015) [38]	12	Ranibizumab ×3 Q4W + PDT → PRN	37	4.5	1.1	62.1	54.3	8.1
		Ranibizumab ×3 Q4W → PRN + deferred PDT	35	6.8	1.4	54.8	54.9	8.8
PLANET (2018) [21]	12	Aflibercept ×3 Q4W → Q8W	145	8.1	0.2 (sham)	38.9	57.7	10.7
		Aflibercept ×3 Q4W → Q8W + rescue PDT	154	8.0	0.2	44.8	59.0	10.8
PLANET (2019) [22]	22	Aflibercept ×3 Q4W → Q8W	137	12.7	0.3 (sham)	82.1	57.7	10.7
		Aflibercept ×3 Q4W → Q8W + rescue PDT	147	12.6	0.3	85.6	59.0	9.1

## Anatomical endpoints and PCV specific biomarkers

In current clinical practice the management of PCV following commencement of initial therapy is guided for the most part by visual acuity, the presence of subretinal and intraretinal fluid, and the status of the PL as mentioned above. While these features have informed the outcomes of previous PCV clinical trials, there is a growing body of literature suggesting several other morphological and physiological features specific to PCV may also have value as treatment biomarkers.

### Polypoidal lesions as biomarkers for treatment

A sharp-peaked PED and a sub-RPE ring-like lesion on OCT have been reported to be frequently associated with and co-localise to PL on ICGA. The Asia Pacific Ophthalmic Imaging Society (APOIS) PCV workgroup has shown that OCT is a viable alternative to ICGA in assessing the presence of PL in treatment naïve eyes as well as in eyes with persistent fluid following monthly anti-VEGF loading treatment [39]. (Fig. 1) Features which suggest presence of PL in eyes with persistent fluid post-treatment include presence of a sharp-peaked PED, a sub-RPE ring on OCT and an orange nodule on colour fundus photography. In addition, the height of the PED as well as reflectivity of the content of the PED may predict PL closure (Tan A.C. et al., manuscript in review). These OCT features can facilitate the assessment of PL perfusion status especially after treatment with less reliance on ICGA. The APOIS PCV workgroup also demonstrated that if deferred PDT is indicated in eyes with persistent fluid, the appropriate PDT spot size and location can be deduced with excellent accuracy using only OCT coupled with near-infrared [40] (100% PL coverage and 90% BNN coverage). Re-examining the baseline scans for an inverted U-shaped elevation in anti-VEGF resistant cases has also been reported to be helpful for detecting PL which might have been overlooked [41].

OCT angiography (OCTA), a novel imaging modality which detects blood flow, may also be able to detect flow signal within the PCV lesion [42, 43]. Specifically, detection of flow within the BNN has been shown to be comparable or even superior to ICGA due to the high contrast available with OCT. However, ICGA may be superior to OCTA for PL identification due to slow PL filling that is not well seen with OCTA. This imaging finding is consistent with histopathological studies which reported that PL are aneurysmal dilatations of neovascular tissue [44], and that the PL arises from the neovascular complex rather than as a primary lesion. The response to anti-VEGF therapy demonstrated in clinical trials discussed in the previous section are compatible with these imaging and histopathological findings [18, 45]. In addition, the nature of the PL based on OCTA features has been debated. While some investigators describe aneurysmal dilatation in the PL, others have suggested the appearance represented tangled vascular structures [44].

### Choroidal features

PCV resides within the pachychoroid spectrum of diseases. These eyes are characterised by a thickened choroid, attenuation of the choriocapillaris and dilated Haller’s layer vessels [46, 47]. Several additional metrics have been used to quantify and grade the characteristics of the choroid; (1) choroidal thickness, (2) choroidal vascular hyperpermeability (CVH), and (3) choroidal vascularity index (CVI). The association of these choroid features with clinical characteristics and treatment outcomes of PCV is currently inconsistent. Moreover, thickening of the choroid is often seen in normal eyes and while it may be more common among PCV patients, it may not form an intrinsic part of PCV pathogenesis.

A thicker choroid was shown to be associated with a poorer anatomical response to initial loading doses of anti-VEGF [48]. Recent work by Chang and Cheng [49] has determined a subfoveal choroidal thickness (SFCT) of 267.5 μm as a useful threshold. Higher SFCT is more than 50% less likely to achieve resolution of SRF within three months of anti-VEGF initiation when compared to normal choroid patients. Choroidal thickness is also positively correlated to PCV lesion area [50] and presumably related to treatment outcomes. On the other hand, a post hoc analysis of imaging biomarkers of the EVEREST II cohort did not show any association between central choroidal thickness and functional or anatomical outcomes [51]. Similarly, CVH and CVI association with treatment outcomes is variable. Some described the favourable outcomes with the presence of CVH with lower number of anti-VEGF treatments combined with PDT [52], while other studies showed that the presence of CVH was associated with poor visual and anatomical outcomes after treatment with anti-VEGF [53, 54].

### Pigment epithelial detachment

Pigment epithelial detachments (PEDs) are a hallmark feature of PCV [55, 56]. PEDs in PCV patients tend to be larger than those in typical nAMD and are associated with a higher incidence of RPE tears but their morphology is not prognostic of visual outcomes at one year [56]. In addition, PEDs, when analysed as a biomarker in any nAMD, were found to be compatible with good vision and did not have a significant impact on visual acuity with treatment [56].

### Novel metrics for quantification for PCV-specific biomarkers

Currently, neither choroidal thickness nor PED have been consistently shown to be associated with outcomes in patients treated for PCV. Most studies have used subfoveal or central choroidal thickness and maximum height or width of PED, or merely the presence or absence of PED. These point measurements and binary grading criteria may not fully encompass the effects of these features on the disease process or activity. With recent advances in segmentation algorithms, volumetric analysis of retinal pathological features such as IRF and SRF have been suggested as useful biomarkers for predicting treatment response [34, 57, 58]. When similarly applied to PED and the choroid, volumetric analysis may provide much more information regarding the impact of these features on the outcomes of PCV. In a post hoc analysis of a PCV monotherapy treatment trial we performed, we found that baseline PED volume, lower CVI and a more rapid reduction in PED volume from baseline to month 3 are associated with reduced disease activity at month 12. These features have yet to be validated as retreatment criteria but show promise in this preliminary work [37].

## Novel therapies on the horizon for PCV

One of the core goals of treatment for neovascular maculopathies is to minimise treatment burden for the patient. There has been considerable effort in the field to either modify the delivery of currently available drugs through less intensive regimens or develop new drugs with the aim of improving treatment durability, efficacy, or safety, compared to currently available agents. While most ongoing registration clinical trials are aimed at nAMD, it is likely that in these cohorts, a subset are PCV. Increasingly, there is interest in investigating the efficacy of these newer agents on PCV.

Brolucizumab is a recently available antibody fragment developed for the management of nAMD and other exudative maculopathies and was found to be similarly efficacious but more durable than aflibercept in the phase 3 HAWK and HARRIER trials [59, 60]. Subgroup analysis of Japanese PCV patients from the HAWK trial showed excellent anatomical and functional outcomes with 8- or 12-weekly injections compared to 8-weekly injections with aflibercept [61]. A recent report by Matsumoto et al. [62] on the 3-month outcomes of type 1 CNV patients (including 19 with PCV) treated with three loading doses of brolucizumab showed polyp regression in 78.9% of cases, combined with impressive visual and anatomical outcomes. Despite these promising findings, the adoption of brolucizumab by retinal physicians had been hampered by reports of intraocular inflammation, in particular occlusive vasculitis, which has in some cases led to permanent visual loss [63].

Another upcoming antibody for nAMD treatment, faricimab, is a bi-specific monoclonal antibody that binds VEGF-A and angiopoietin-2 (Ang-2), the latter being up-regulated in neovascular membranes and promoting vascular leakage in combination with VEGF-A [64, 65]. Recently released data from the phase 3 TENAYA and LUCERNE studies [66] have demonstrated that faricimab is non-inferior to aflibercept in terms of functional and structural outcomes in nAMD patients, but can be used with longer treatment intervals of up to 16 weeks, compared to every 8 weeks for patients treated with aflibercept. There is no report of the efficacy and safety of faricimab on PCV yet. The additional blockage of Ang-2 has been proposed to promote vascular stability. Whether this translates into any significant anatomical advantage in typical nAMD or PCV remains to be seen.

An alternative to regular intravitreal injections for long-term nAMD treatment is the surgically implanted port delivery system (PDS) for ranibizumab [67]. The PDS is a small refillable reservoir for an anti-VEGF antibody that is surgically implanted at the pars plana via scleral cutdown and can be periodically refilled via direct injection through the conjunctiva. A PDS loaded with 100 mg/mL ranibizumab had a median reload time of 15 months but with comparable efficacy to monthly ranibizumab injections in the phase 2 LADDER trial [67]. The real-world safety profile of the PDS remains to be seen, but at present it shows promise for patients in need of long-term anti-VEGF therapy who wish to avoid frequent injections. There is no study of the PDS in eyes with PCV to-date.

## Future research directions

### Developing PCV-specific biomarkers

Heterogeneity among PCV lesion characteristics and treatment responses is well recognised. In EVEREST II better visual gains were achieved, somewhat predictably, among those with younger age, poorer baseline vision, and smaller PCV lesion size for patients in the ranibizumab monotherapy arm [51]. Higher vision gains are associated with higher anti-VEGF injection frequency [4], CVH [52], and thinner subfoveal choroid [48], while poorer visual outcomes have been reported in cases with fibrovascular PEDs [68] or subretinal fibrosis [69], and sub macular haemorrhage [70]. Furthermore, in the EVEREST II study, a subgroup of patients (~30%) in the combination arm required only 3–4 intravitreal ranibizumab injections over 2 years [20]. However, there is currently no reliable method to prospectively identify this subgroup. Advanced image analysis tools and characterization of PCV-specific biomarkers beyond those recognised in typical nAMD will further develop personalisation of PCV therapies.

### Understand phenotypes associated with poor vision outcome- fibrosis and macular atrophy

The end-stage phenotypes of nAMD are well described and can represent a stage of disease in which treatment is often futile. Both fibrosis and macular atrophy have been associated with various baseline clinical and treatment features such as nAMD subtype, visual acuity and frequency of treatment. [45, 71–75] These phenotypes, however, are not well understood in the PCV subtype. Incident fibrosis was studied in an Asian population with about 48% of the cohort identified as having PCV [69]. There was a threefold increase in incident fibrosis (from 10% noted at baseline to about 30% at 12 months), but no difference in the rate of subretinal fibrosis between PCV and typical nAMD. The rate of macular atrophy was found to be lower than prior reports. Taken together, these findings suggest that fibrosis was a more significant end stage phenotype than macular atrophy in Asian (PCV) patients [69]. Further understanding of the developmental differences of fibrosis between PCV and typical nAMD, as well as molecular pathways involved in the development of fibrosis, are necessary and offer opportunities for targeted therapies against these phenotypes.

### Long-term recurrence

In typical nAMD, reports of up to 10-year outcomes are available [76, 77]. In contrast, currently available clinical trial data for PCV mostly report visual improvements in the range of 5–10 letter gain at 2 years [20, 22]. A small number of real-world retrospective reports on the 5-year outcomes for PCV patients suggest that visual outcomes and treatment burdens are similar to typical nAMD [78]. In PCV eyes, however, there appears to be a distinct period of lesion reactivation that occurs at around 2–3 years after initial presentation that requires reversion to a more intensive treatment regimen [79]. Previous work from Kim et al. [80] suggests that the presence of clusters of multiple PL and larger lesion size are associated with PCV reactivation during the first year, though it is unclear what features dictate reactivation in the long-term and such data would be valuable when formulating management strategies for patients [81]. In addition, data on long-term incidence of submacular haemorrhage in PCV is lacking. A recent study found that submacular haemorrhage occurred in 10% in 5 years and 30% at 10 years. This rate is significantly higher than that of typical nAMD, reported to occur in only 4% in 5 years and 10% in 10 years [82]. The presence of a cluster of polyps was found to be significantly associated with submacular haemorrhage, further supporting the importance of PL status as a treatment endpoint for PCV.

### Addressing choroidal background

Therapies that directly address choroidal congestion may be effective for PCV treatment due to the strong evidence for choroidal features playing a role in the pathophysiology of PCV. Currently, PDT offers the most potential as a therapeutic option that targets the choroid. Some studies have demonstrated a reduction in CVH and choroidal thickness after PDT [83–85]. Further evidence of the effects of PDT on the choroid has been proposed in the form of modulation of large choroidal vessels in the Haller’s layer. However, this effect appears short-lived and the dilated vessels return to their original configuration eventually [86]. Taken together, these observations suggest that current therapies reduce the leakiness of the choroid but effects may be transient as the dilated vessels may be an irreversible feature resulting from chronic structural remodelling and the formation of anastomoses [87, 88]. These features may not be easily reversed by pharmacological means and further research into their mechanism of formation is required.

### Upstream mediators in PCV pathogenesis

Most current PCV treatment strategies still hinge on the role of VEGF as a mediator of choroidal neovascularization and vascular permeability, a role which has been appreciated for 25 years [89]. More recent work has focused on other physiological mediators that may play a role in PCV pathogenesis. Work by Jones et al. [90] and Kumar et al. [91] showed that the serine protease HtrA1, when overexpressed in transgenic mice, was sufficient to induce cardinal features of PCV including a branching network of choroidal vessels and PL via a mechanism involving thinning of Bruch’s membrane and VEGF stimulation. Several components of the lipid metabolome have been linked to PCV through epidemiologic, genetic and biochemical studies [92–94]. Metabolomic studies have implicated several oxidised lipids and amino acids, including hyodeoxycholic acid and L-tryptophanamide, as retinal epithelial modulators with differential abundance in PCV compared to typical nAMD [95]. Both species influenced apoptosis and necrosis in cultured RPE and retinal endothelial cells. A synergy between oxidised lipids and HtrA1 to promote VEGF expression and ultimately stimulate neovascular membrane development was recently appreciated [96]. Anti-HtrA1 antibodies are already under investigation for management of geographic atrophy [97], though it remains to be seen if this class of drugs also have utility in the treatment of PCV.

## Conclusions

This review demonstrates how the understanding of PCV has evolved with imaging studies and therapeutic trials. These advances have in turn translated into treatments, which promote sustained functional outcome and are aligned with the pathophysiology of the condition. There remains significant heterogeneity among retinal physicians in terms of preferred PCV treatment approaches, although clinical outcomes have improved substantially since the initial discovery of PCV. Recurrence of the disease 2–3 years after successful treatment is still a major concern. Future work which addresses the unique aspects of this condition will no doubt provide additional treatment options specifically tailored for PCV in the form of expanded drug choice and alternative dosing regimens, likely with a strong focus on improved treatment durability and reduced treatment burden.
